# Clarifying Blood Indices in Patients With Ovarian Cancer

**DOI:** 10.2196/70895

**Published:** 2025-08-13

**Authors:** Chengwei Lian, Yu Fan, Jinke Li

**Affiliations:** 1 Department of Gynaecology and Obstetrics, West China Second Hospital, Sichuan University, No 20, Sec 3, Renminnan Road, Sichuan 610041, Chengdu, China, 86 28-85502391; 2 Key Laboratory of Birth Defects and Related Diseases of Women and Children, Sichuan University, Ministry of Education, Chengdu, China

**Keywords:** WeChat, nutrition management, ovarian cancer, chemotherapy, mobile health

This letter is regarding the recent publication “Efficacy of a WeChat-Based, Multidisciplinary, Full-Course Nutritional Management Program on the Nutritional Status of Patients With Ovarian Cancer Undergoing Chemotherapy: Randomized Controlled Trial” [1]. The authors reported a novel approach to managing the nutrition status of patients with ovarian cancer who underwent chemotherapy. With supportive results, it might be a potentially beneficial program for this patient cohort. However, there might be a problem in the description of Figure 7.

The authors described the changes in leukocytes, lymphocytes, neutrophils, and platelets as “inflammation-related blood indices.” It might be appropriate for most patients with normal immune status but is not applicable for those undergoing chemotherapy with bone marrow suppression (BMS) [2]. BMS is one of the most common adverse effects in patients undergoing chemotherapy and can be divided into chemotherapy-induced leukopenia, neutropenia, thrombocytopenia, and anemia [3]. The changes in the blood cells above are not only an indication of inflammatory status but also critical to monitor the BMS in patients. It is important to discuss BMS in treatment using chemotherapy separately or combined with others. Additionally, the authors have reported a systematic inflammatory index, which is more accurate, to evaluate the inflammatory status of patients [4]. As for Figure 7, we noticed a significant decrease in leukocytes, dropping almost to the diagnostic criteria of leukopenia [5] in the control group, which suggests more severe BMS in the control group. So we suggest it might be more accurate to describe Figure 7 simply as “the changes in blood cells,” and the potential benefit for improving BMS should also be described in this section.

The findings of this study are meaningful and interesting. The authors provided a potential approach to improve the clinical outcomes of patients with ovarian cancer undergoing chemotherapy, especially in China. Additionally, we noticed the intervention could improve BMS for these patients, although it should be described and addressed more accurately in the publication.

## Abbreviations

BMS: bone marrow suppression
